# Alzheimer’s disease and progressive supranuclear palsy share similar transcriptomic changes in distinct brain regions

**DOI:** 10.1172/JCI149904

**Published:** 2022-01-18

**Authors:** Xue Wang, Mariet Allen, Özkan İş, Joseph S. Reddy, Frederick Q. Tutor-New, Monica Castanedes Casey, Minerva M. Carrasquillo, Stephanie R. Oatman, Yuhao Min, Yan W. Asmann, Cory Funk, Thuy Nguyen, Charlotte C.G. Ho, Kimberly G. Malphrus, Nicholas T. Seyfried, Allan I. Levey, Steven G. Younkin, Melissa E. Murray, Dennis W. Dickson, Nathan D. Price, Todd E. Golde, Nilüfer Ertekin-Taner

**Affiliations:** 1 Department of Quantitative Health Sciences and; 2 Department of Neuroscience, Mayo Clinic Florida, Jacksonville, Florida, USA.; 3 Institute for Systems Biology, Seattle, Washington, USA.; 4 Department of Biochemistry and; 5 Department of Neurology, Emory University, Atlanta, Georgia, USA.; 6 Department of Neuroscience, Center for Translational Research in Neurodegenerative Disease, McKnight Brain Institute, University of Florida, Gainesville, Florida, USA.; 7 Department of Neurology, Mayo Clinic Florida, Jacksonville, Florida, USA.

**Keywords:** Aging, Neuroscience, Alzheimer disease, Bioinformatics

## Abstract

Vast numbers of differentially expressed genes and perturbed networks have been identified in Alzheimer’s disease (AD), however, neither disease nor brain region specificity of these transcriptome alterations has been explored. Using RNA-Seq data from 231 temporal cortex and 224 cerebellum samples from patients with AD and progressive supranuclear palsy (PSP), a tauopathy, we identified a striking correlation in the directionality and magnitude of gene expression changes between these 2 neurodegenerative proteinopathies. Further, the transcriptomic changes in AD and PSP brains ware highly conserved between the temporal and cerebellar cortices, indicating that highly similar transcriptional changes occur in pathologically affected and grossly less affected, albeit functionally connected, areas of the brain. Shared up- or downregulated genes in AD and PSP are enriched in biological pathways. Many of these genes also have concordant protein changes and evidence of epigenetic control. These conserved transcriptomic alterations of 2 distinct proteinopathies in brain regions with and without significant gross neuropathology have broad implications. AD and other neurodegenerative diseases are likely characterized by common disease or compensatory pathways with widespread perturbations in the whole brain. These findings can be leveraged to develop multifaceted therapies and biomarkers that address these common, complex, and ubiquitous molecular alterations in neurodegenerative diseases.

## Graphical abstract

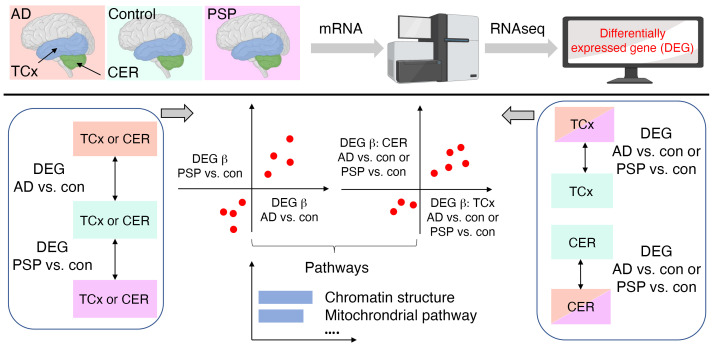

## Introduction

Neurodegenerative proteinopathies such as Alzheimer’s disease (AD) and progressive supranuclear palsy (PSP) are characterized by the aggregation and accumulation of self-proteins within insoluble aggregates (1). AD is a complex proteinopathy characterized by extracellular amyloid β (Aβ) protein deposits and intracellular neurofibrillary tangles (NFTs) composed of the microtubule-associated protein tau (2). In PSP, which is considered a pure tauopathy, tau pathology is observed in several cell types. Tau accumulates as NFTs in neurons, as “tufts” in astrocytes (hence, the descriptor “tufted astrocytes”), and in coiled bodies or glial inclusions in oligodendrocytes (3). In both diseases, numerous lines of research show a strong link between protein aggregation, accumulation, and degeneration, although the precise mechanisms of cellular dysfunction and death remain enigmatic. Indeed, there is little consensus as to the mechanisms underlying cell dysfunction and death in AD, PSP, and other neurodegenerative proteinopathies. Because of this incomplete understanding, multiple studies are now using system-level omics approaches to further understand the pathological cascades in AD, PSP, and other neurodegenerative proteinopathies (4–6). Here, we compared the transcriptomic changes in 2 brain regions from a large series of postmortem AD, PSP, and control brain samples.

## Results and Discussion

### Transcriptomic changes are conserved between AD and PSP.

**Figure 1:**
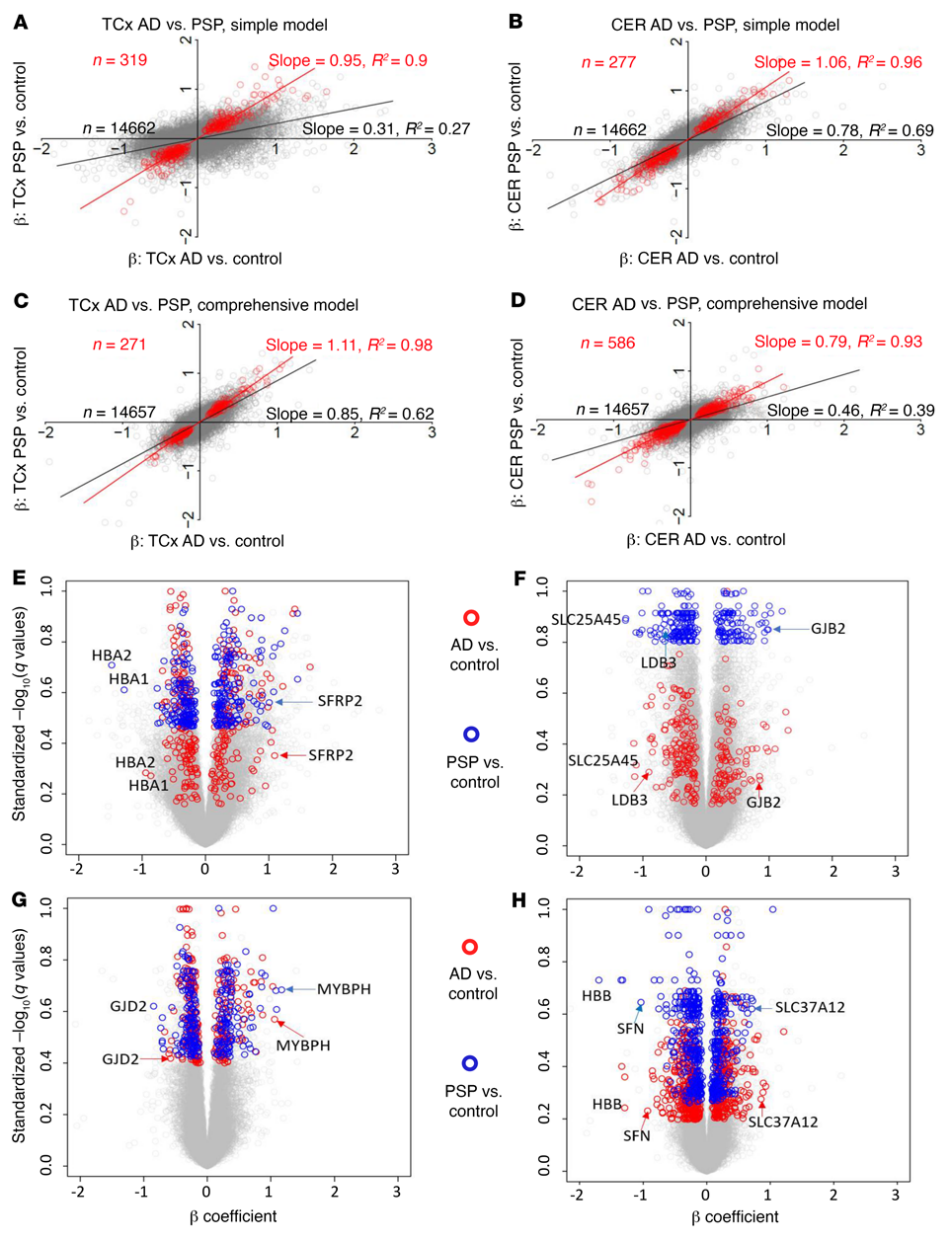
Gene expression changes. (**A**–**D**) Comparison between β coefficients (β) of AD versus control and those of PSP versus control DEG analyses. Each circle represents a gene. Simple model: β was derived from linear regression, with expression as the dependent variable, diagnosis as the independent variable of primary interest, and RIN, age at death, sex, source of samples, and flowcell as covariates. Comprehensive model: β was derived from linear regression as in the simple model, with expression of 5 cell type markers as additional covariates. Red circles: DEGs with *q* < 0.05 on both side comparisons, except for in **D**, where *P* < 0.05 was used in CER PSP versus control analyses. (**E**–**H**) Volcano plots highlighting genes from **A**–**D**, respectively. The analysis included 231 TCx and 224 CER samples.

**Figure 2:**
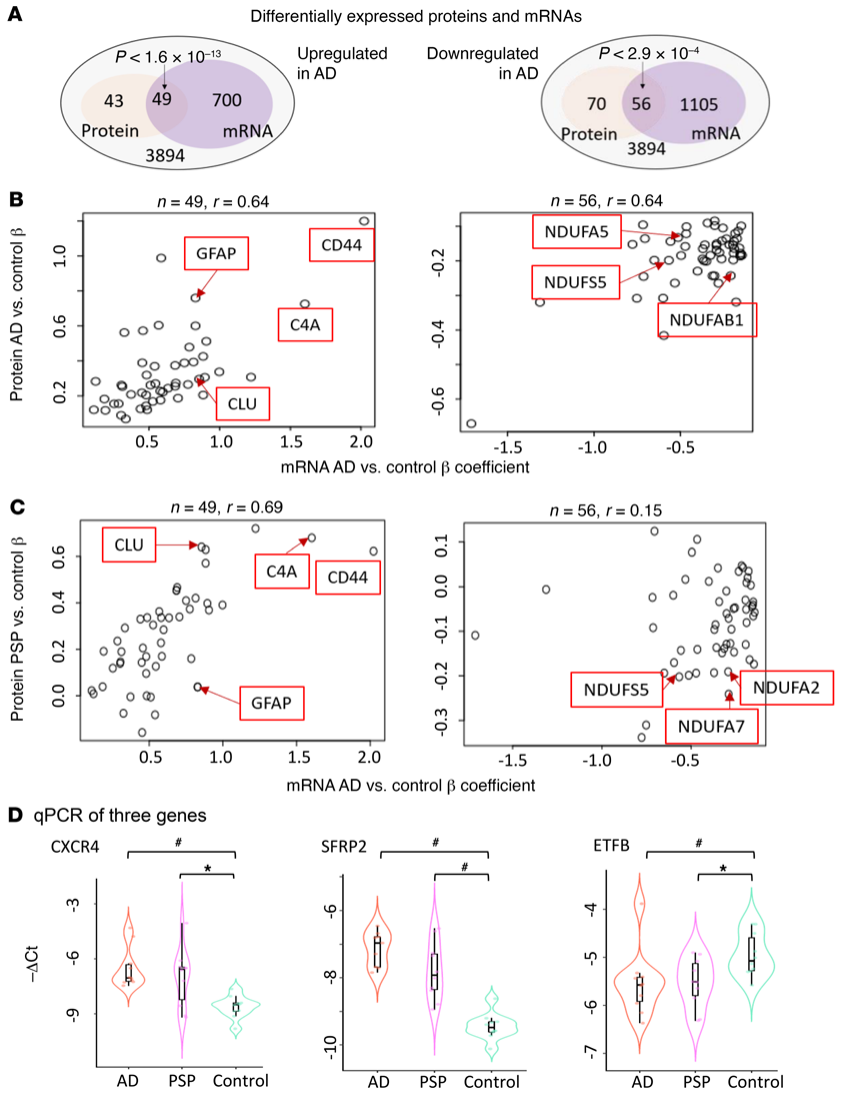
Protein and qPCR validation of differentially expressed genes. (**A**) Venn diagram of proteins and genes that were differentially expressed at an FDR of 0.05 between AD and control samples. Overrepresentation *P* values were from a hypergeometric test. (**B** and **C**) Scatter plot of the overlapping upregulated or downregulated proteins and genes identified in **A**. (**B**) AD vs. control DEG β coefficients are plotted against AD vs. control protein β coefficients. (**C**) PSP vs. control DEG β coefficients are plotted against AD vs. control protein β coefficients. (**D**) qPCR results of *CXCR4*, *SFRP2* and *ETFB*. *n* = 10 samples in each diagnosis group. *P < 0.05, by 1-sided Wilcoxon rank-sum test; ^#^*P* < 0.05, by 1-sided Wilcoxon rank-sum test with Bonferroni correction.

We compared the change in gene expression between AD and control and PSP and control in the temporal cortex (TCx) and cerebellar cortex (CER) (5, 7). Supplemental Table 1 indicates the samples and data used. At a genome-wide level, we analyzed the data using 2 linear regression models (5). First, we used a simple model, in which differential gene expression was assessed using linear regression, with expression as the dependent variable, diagnosis as the independent variable of primary interest, and RNA integrity number (RIN), age, sex, source of samples, and flowcell as covariates (Supplemental Tables 2–5; supplemental material available online with this article; https://doi.org/10.1172/JCI149904DS1). Second, we applied a comprehensive model to partially account for cell type changes (Supplemental Tables 6–9). In the comprehensive model, we used the expression of 5 genes that serve as cell type markers (*ENO2* for neurons, *CD68* for microglia, *OLIG2* for oligodendrocytes, *GFAP* for astrocytes, and *CD34* for endothelial cells) as covariates, in addition to all covariates in the simple model (7). These 2 models are described in the Supplemental Methods and in our previous publication (5). For the analyses described here, we filtered the TCx and CER data for protein-coding genes detected in both data sets above background on the basis of their conditional quantile-normalized values (5). This filtering resulted in the identification of 14,662 common genes in the TCx and CER with associated β coefficients and *q* values of differential expression (DE) between AD and the control and PSP and the control. For our comprehensive model analyses, this number was 14,557 because of the exclusion of 5 cell type marker genes. Supplemental Table 1 shows the summary data for the differentially expressed genes (DEGs), revealing large-scale transcriptomic changes in the protein-coding transcriptome for the AD TCx and CER, with fewer DEGs withstanding false discovery in the PSP TCx and CER. Using these data, we generated plots of the β coefficients of AD versus control (*x* axis) and PSP versus control (*y* axis) DE, using either no additional filter or filtering for various *q* value (i.e., FDR-adjusted *P* value) cutoffs. Even when examining all genes without a DEG *q* value filter, we found a strong positive correlation between the changes observed in AD versus control and PSP versus control TCx and CER samples (Figure 1, A and B). Assessment of the data from the simple model for all genes using linear regression revealed an *R2* of 0.27 (Figure 1, A and E; *P <* 1.0 × 10^–10^; slope, 0.31) for the TCx and an *R2* of 0.69 (Figure 1, B and F; *P <* 1.0 × 10^–10^; slope, 0.78) for the CER. These *R2* values were increased and remained highly significant when analyzed using the comprehensive model. In the TCx, the *R2* was 0.62 (Figure 1, C and G; *P <* 1.0 × 10^–10^; slope, 0.85), and in the CER, the *R2* was 0.39 (Figure 1, D and H; *P <* 1.0 × 10^–10^; slope, 0.46). In both models, increasing the *q* value cutoff to 0.1, 0.05, or 0.01 reduced the number of genes but increased the strength of the correlations, with *R2* ranging from 0.89 to 0.98 and slopes ranging from 0.77 to 1.13 (Supplemental Table 10). We also illustrate the conserved gene expression changes using heatmaps (Supplemental Figures 1 and 2) and volcano plots Figure 1, E–H). To validate our findings, we also analyzed proteomics data (Figure 2, A–C) and performed quantitative PCR (qPCR) on 3 select genes (*CXCR4*, *SFRP2*, and *ETFB)* (Figure 2D) and immunohistochemical analyses on 2 of these with suitable antibodies (CXCR4, ETFB; Supplemental Figures 3 and 4). Using the proteomics data, we identified significant overlap between genes and proteins that changed in the same direction in AD brains compared with control brains, validating the transcriptomic changes in the AD brains. These validated genes also had concordant transcript changes in PSP brains, but their protein levels were not significantly perturbed in PSP. Our qPCR data independently validated the RNA-Seq results for the selected genes, and immunohistochemistry demonstrated their localizations.

These analyses show a striking conservation in the overall patterns of gene expression in 2 neurodegenerative disorders in 2 regions of the brain. These regions at the level of visible and gross pathologies are quite distinct. The TCx is severely affected in AD (8). It is atrophied, with prominent neuronal synaptic loss and shows robust amyloid and tau pathologies and gliosis. In PSP, TCx tau pathology and neuronal loss are less severe than what is observed in AD and even other regions of the brain affected earlier in the PSP disease course (9). In contrast, the CER is not typically reported to be pathologically affected in either AD or PSP, although certainly in PSP, deep cerebellar nuclei are affected. Nonetheless, connections between the CER and brain areas may be damaged by both disorders (10). Both the overall correlations in the entire set of genes analyzed and the increasing correlations observed when a *q* value filter was applied demonstrated that the transcriptomic changes for protein-coding genes were highly similar in these 2 disorders, and that DEGs selected on the basis of *q* value cutoffs represented the core transcriptomic changes observed during neurodegeneration. Further, as bulk RNA-Seq data from whole-brain tissue is strongly influenced by changes in cell-type composition (11), we noted that the comprehensive model that takes into account these cell type changes had a stronger correlation in the TCx between the disease states when compared with the simple model when no *q* value cutoff was used. As the CER is relatively unaffected in terms of alterations in cell type composition, when all genes were analyzed, the correlation was actually weaker. Once a *q* value filter was applied, we observed little difference between the models. Such data indicate that cell type changes indeed contributed to some of the transcriptome variance observed, and correcting for that variance in the bulk RNA-Seq data could increase the power of the study to detect DEGs replicably across neurodegenerative diseases, when a tissue has cell type changes in 1 or both conditions, but may impair analyses when no large-scale cell type changes are present.

### Transcriptomic changes are conserved across the TCx and the CER.

**Figure 3:**
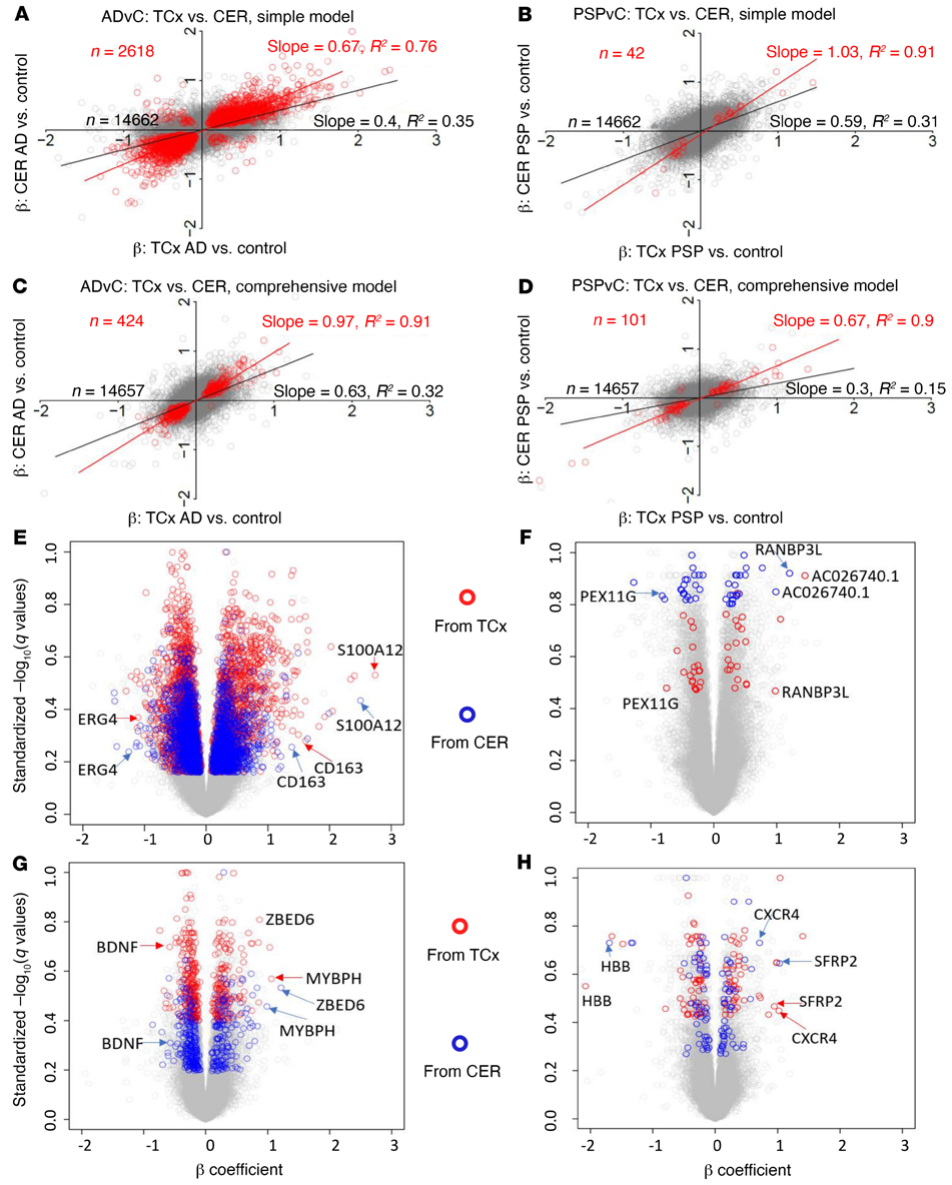
Gene expression changes are conserved between brain regions within disease analyses. (**A**–**D**) Comparison between β coefficients of TCx AD versus control (ADvC) and those of CER ADvC, and between TCX PSPvC and CER PSPvC DEG analyses. Red circles indicate DEGs with *q* < 0.05 on both side comparisons, except for in **D**, where *P* < 0.05 was used for PSPvC. Simple model: β was derived from linear regression with expression as the dependent variable, diagnosis as the independent variable of primary interest, and RIN, age at death, sex, source of samples, and flowcell as covariates. Comprehensive model: β was derived from linear regression as in the simple model, with the expression of 5 cell type markers as additional covariates. (**E**–**H**) Volcano plots highlighting genes from **A**–**D**, respectively. The analysis included 231 TCx and 224 CER samples.

The DEG changes between AD and PSP in 2 regions of the brain demonstrate a striking conservation of transcriptomic changes across these different neurodegenerative diseases. In designing these studies, we considered the CER as an internal control for a relatively unaffected area of the brain. However, given the large number of highly significant DEGs in the AD CER, we evaluated whether the transcriptomic changes in the TCx and CER were also conserved within a disease classification (Figure 3). In this case, we plotted the β coefficients for AD versus control in the TCx (*x* axis) versus the β coefficients for AD versus control in the CER (*y* axis) and likewise generated plots of the β coefficients of the TCx versus the CER for PSP versus control. We plotted data from both the simple and comprehensive models. These analyses showed robust correlations. In AD, the overall *R2* between the TCx and the CER was 0.35 (Figure 3, A and E; *P <* 1.0 × 10^–10^; slope, 0.40) using the simple model, and the *R2* was 0.32 (Figure 3, C and G; *P <* 1.0 × 10^–10^; slope, 0.63) using the comprehensive model. In PSP, the overall *R2* was 0.31 (Figure 3, B and F; *P <* 1.0 × 10^–10^; slope, 0.59) in the simple model, and the *R2* was 0.15 (Figure 3, D and H; *P <* 1.0 × 10^–10^; slope, 0.3) in the comprehensive model. Again, as the stringency of the *q* value used to select the DEGs was increased, both *R2* (range, 0.70–0.95) and the slope (range, 0.62–1.03) of the best-fit line increased when comparing the transcriptomes for the TCx and CER within disease states (Supplemental Table 10). Thus, not only were the transcriptomic changes conserved between AD and PSP, they were also conserved across a severely affected and “unaffected” brain region in AD and a moderately affected and less-affected brain region in PSP. We have also illustrated the conserved gene expression changes using volcano plots (Figure 3, E–H).

### Gene ontology analyses.

**Figure 4:**
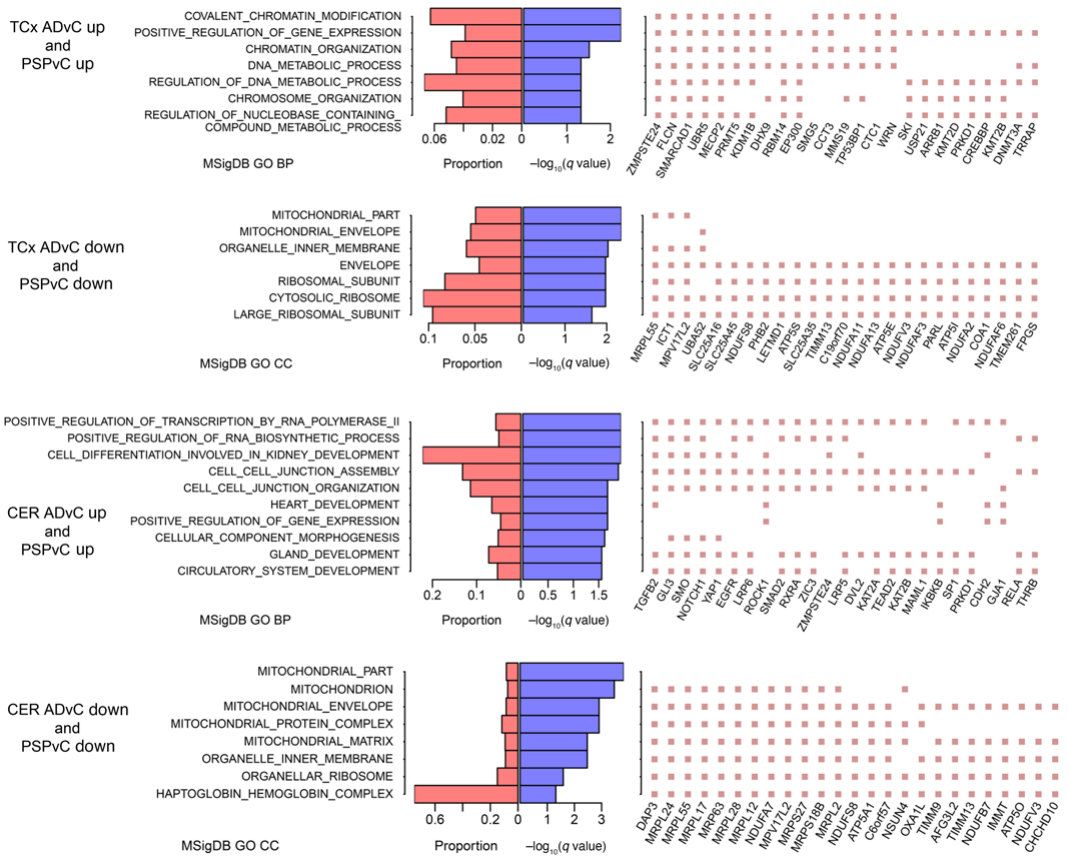
GO enrichment of DEGs. Left panel: GO biological process (BP) terms of enrichment (*q* < 0.05) are listed; when no such BP or molecular function term existed, cellular compartment (CC) terms of enrichment (*q* < 0.05) are listed. Middle panel: –log_10_ enrichment *q* value (purple bars) and proportion of DEGs in GO term over GO term genes (red bars). Right panel: top 25 DEGs that were mostly observed in the selected GO terms. DEGs were identified at *q* < 0.1 in both AD versus control and PSP versus control comparisons. Up, upregulated; down, downregulated; MSigDB, Molecular Signatures Database.

Given these striking correlations of DEG changes across 2 neurodegenerative disorders and 2 brain regions, we used gene ontology (GO) analyses to provide some biological context to these data. In this case, we binned the input into the GO analyses by focusing on DEGs (*q* < 0.1) that were changed in the same direction. Thus, we first analyzed DEGs that were downregulated in AD and PSP or upregulated in AD and PSP using the Functional Mapping and Annotation (FUMA) GWAS web server (12). These data are summarized in Figure 4, with more detailed versions provided in Supplemental Tables 12–17. Shared upregulated DEGs in the TCx of AD and PSP brains were enriched (enrichment *q* < 0.05) for biologic processes related to chromatin modification, gene expression, chromosome organization, and metabolism of nucleotides. In the CER, the shared upregulated genes linked to biological processes relating to RNA and RNA transcription, cell-cell junctions, and heart, kidney, gland, and circulatory system development. Shared downregulated genes in AD and PSP were associated with GO cell compartment terms related to mitochondrial and ribosomal functions in both the TCx and the CER. These data and the extended GO analyses (Supplemental Tables 12–17) point to highly complex biological changes shared in both AD and PSP. Epigenetic modifications constitute 1 type of mechanism that may drive some of these transcriptional changes in AD and PSP brains. Using available assay for transposase-accessible chromatin using sequencing (ATAC-Seq) (13), histone acetylation (14), and methylation (15) data, we determined that many of the genes in Figure 4 and Supplemental Tables 12–17 are under epigenetic control (Supplemental Figure 5 and Supplemental Tables 18–22).

## Discussion

Numerous studies analyzing large-scale transcriptomic alterations in AD have revealed a large number of network abnormalities that demonstrate widespread changes in pathways including but not limited to immune function, myelination, synaptic transmission, and lipid metabolism (4, 5, 11, 16–19). Although these postmortem cross-sectional data sets provide a detailed systems-level description of changes that have occurred over the disease course, in isolation they do not provide a framework for cause-and-effect relationships. The conservation in the overall transcriptome signature of AD and PSP relative to control brains indicates that the transcriptomic changes observed are more likely attributable to common downstream events in the neurodegenerative cascade and not initiating events. The fact that these conserved transcriptomic changes were observed in regions with neuropathologies varying from minimal to significant suggests that these conserved expression changes are unlikely to be driven by gross neuropathology or cell proportion changes. We have previously identified reduced expression of myelination network transcripts and proteins in both AD and PSP TCx and nominated it as a common disease mechanism for both conditions (5). Given that AD and PSP are both tauopathies, conserved transcriptional alterations may not generalize to all neurodegenerative disorders. That said, the conservation holds in the CER, which is thought to be largely unaffected in these disorders, and therefore we would speculate that carefully conducted transcriptomic studies that are expanded to include other neurodegenerative proteinopathies may well show similar shared transcriptomic changes reflecting a long-standing neurodegenerative process triggered by protein accumulation.

Our finding that there were shared transcriptomic changes between the TCx and the CER in AD and PSP brains is noteworthy and consistent with our prior findings in transcriptional networks (5). As noted previously, we had intended the CER to serve as a “control” for a largely pathologically unaffected brain region in AD; however, these transcriptomic data indicated a strong correlation between DEGs in both regions. Although this correlation was more robust because of the larger number of DEGs in AD versus control brains, the correlation held in PSP. This observation has several implications. First, these data demonstrate that long-standing neurodegenerative disease processes have a broad impact on the brain that extends well beyond visible pathology. Thus, there needs to be appropriate caution when inferring that a brain region in disease is “unaffected” based on an absence of pathological abnormalities as assessed using standard methods. Second, highly similar transcriptomic alterations in the brain driven by a regional or multiregional proteinopathy likely reflect a mixture of common degenerative and compensatory responses attributable to long-standing pathology within the brain, such as dysregulations of mitochondria (20).

In summary, the concept that AD, PSP, or any other neurodegenerative disease has a specific transcriptomic signature may be inaccurate; rather, there appears to be conserved transcriptomic alterations due to common proteinopathies or their downstream effects. This assertion will require additional large-scale transcriptomic analyses of other age-associated neurodegenerative diseases conducted in a manner that eliminates many of the experimental confounds, such as batch effects. The large number of highly perturbed networks in AD that have been established in prior studies and our analyses in this study reinforce the notion that, in the symptomatic phase, neurodegenerative diseases are characterized by incredibly complex biology that likely represents a mix of long-standing degenerative and compensatory processes. Such data reinforce the need to both develop paradigms that allow for the earliest possible intervention in these disorders that typically have long prodromal phases, and to develop multifaceted therapies that might be able to better alter the complex alterations present in the symptomatic phases of disease. Our findings also demonstrate the widespread perturbations of systems in the whole brain in neurodegenerative diseases, which requires novel biomarkers capable of tracking these changes in relatively “unaffected” brain regions and formulating therapies that address these ubiquitous alterations.

## Methods

Additional information can be found in Supplemental Results and Supplemental Methods.

### Data availability.

The access information for the TCx and CER transcriptomic and proteomics data is provided in Supplemental Table 1. The access information for the epigenomic data sets is provided in Supplemental Table 18.

### Study approval.

This study was approved by the Mayo Clinic IRB. Informed consent was obtained from the participants or their next-of-kin as applicable.

## Author contributions

TEG, NET, and MA conceived the idea. XW, MA, JSR, YM, and SRO performed quality control and analyses. NET, TEG, MA, CF, and NDP developed the study concept. TEG, XW, and NET wrote the manuscript. DWD provided tissue from the Mayo Clinic Brain Bank and neuropathologically characterized the Mayo brain samples. NTS and AIL generated the proteomics data. TN, KGM, MA, and MMC performed sample selection and library preparation. OI, FQTN, and CCGH performed qPCR experiments. MCC and MEM performed immunohistochemistry experiments. TEG, NET, SGY, and YWA supervised the analytical aspects of the project. NET and TEG provided funding, supervision, and direction for the whole study. All authors read and approved the final manuscript.

## Supplementary Material

Supplemental data

Supplemental tables 1-10

Supplemental tables 11-22

## Version 1 11/23/2021

In-Press Preview

## Version 2 01/18/2022

Electronic publication
