# Supplementary material for: Alzheimer’s disease and progressive supranuclear palsy share similar transcriptomic changes in distinct brain regions
Source: J Clin Invest. 2022 Jan 18;132(2):e149904. doi: 10.1172/JCI149904 (PMC8759790; doi:10.1172/JCI149904)
Supplement: Supplemental data [file jci-132-149904-s029.pdf]

Table of Contents

Supplemental Results 2

Heatmaps and volcano plots of highlighted genes in main Figures 1 and 2 2

Proteomics Validation 2

qPCR validation of *CXCR4, SFRP2* and *ETFB* 3

IHC experiment of CXCR4 and ETFB 3

Analysis of genes in Figure 3 using public epigenetics datasets 4

Evidence of epigenetic modification for Figure 3 genes 4

Evidence of glial broad promoter ATAC-seq peaks for Figure 3 genes 5

Evidence of histone modifications for Figure 3 genes in AD 5

Methods 6

Subjects and Samples 6

RNA sequencing 6

Regression analysis 7

GO enrichment analysis 7

Heatmaps and Volcano Plots 7

Proteomics 8

Validation of *CXCR4, SFRP2* and *ETFB* expression 8

IHC experiments of CXCR4 and ETFB 8

ATAC-seq peak analysis 9

H3K27ac, H3K9ac and H3K122ac analysis 9

Methylation analysis 10

Study approval 10

Data access 10

Acknowledgements 11

References 13

Supplementary Figures 14

Supplemental Results

Heatmaps and volcano plots of highlighted genes in main Figures 1 and 2

To further visualize the highlighted genes in main Figure 1A-D and Figure 2A-D, we plotted heatmaps of scaled normalized gene expression averaged per group. As shown in supplementary Figure S1, genes corresponding to Figure 1A-D show similar patterns between AD and PSP groups which are distinct from control group in both TCx and CER regions. Similarly, in supplementary Figure S1 we observe that, the expression pattern of genes corresponding to main Figure 2A-D are similar in AD and PSP between different brain regions but are distinct from control group. In addition, we made volcano plots for highlighted genes in main Figure 1A-D and Figure 2A-D. As supplementary Figure 1E-H and Figure 2E-H indicate, the β coefficients between AD vs control and PSP vs control are comparable, confirming that both the directionality and magnitude of changes for highlighted genes in main Figure 1 and 2 are conserved.

Proteomics Validation

We downloaded a proteomics dataset containing differential protein expression results between AD and controls for ~5000 peptides/proteins. Of note, 80 AD and 25 control subjects included in the proteomics study are also in this transcriptome study. We determined significant overlap (Figure 4A) of proteins and mRNAs that are up in AD vs. controls (49 genes, $p<1.6E-13$) and those that are down (56 genes, $p<4.8E-04$). The Pearson correlation of beta coefficient between protein and mRNA of these genes is ~0.64 (Figure 4B). Thus, despite the much smaller number of peptides measured compared to the transcriptome, there were concordant protein changes of these transcriptome data. Most of these validated transcripts changed in the consistent direction in PSP vs. controls (beta of the up and down transcripts between AD and PSP, Figure 4C). Amongst these concordantly perturbed transcripts with protein validation are astrocytic molecules such as CLU and CD44 that are expressed at higher levels in AD and PSP and mitochondrial NDUF family genes that are down in both neurodegenerative diseases. Proteome data was also available on TCx for PSP. Most of these proteins were not significantly different in PSP compared to control brains, possibly due to the lack of proteome changes in PSP brains despite transcriptome changes. In conclusion, we identified transcripts that are similarly up or down regulated in both AD and PSP, with proteome validation in a sizable cohort of AD brains.

qPCR validation of *CXCR4, SFRP2* and *ETFB*

Using our RNAseq data, we selected three genes with significant and concordant changes in AD and PSP brains, namely *CXCR4*, *SFRP2* and *ETFB* for qPCR validations of these RNAseq data. As illustrated in Figure 4D and summarized in Table S11, qPCR confirmed that the mRNA expressions of *CXCR4* and *SFRP2* are significantly increased in both AD and PSP in TCx whereas *ETFB* mRNA expression is significantly decreased in AD and PSP in TCx (one-sided Wilcoxon rank-sum test).

CXCR4 is a chemokine receptor and in CNS mostly expressed in microglia. A variant near it is associated with increased risk of multiple neurodegenerative diseases[1]. In tau mouse models, *CXCR4* expression is dysregulated in brain regions that accumulate neurofibrillary tangles[1]. According to our RNAseq transcriptome data of CER, *CXCR4* expression is significantly higher in both AD and PSP brains. On TCx, *CXCR4* expression is significantly higher (FDR=4.3E-03) in AD; it is also higher in PSP although it does not reach the FDR cutoff (p-value=9.5E-03, FDR=0.15). SFRP2 is involved in Wnt-singling pathway, energy metabolism and extracellular remodeling[2]. It has been shown that in mouse adult brain, Sfrps interfere with ADAM10-regulated APP processing[3]. According to our RNAseq transcriptome data on TCx, *SFRP2* expression is significantly up in both AD and PSP when compared to controls. The third gene we validated through qPCR is *ETFB*, which is involved in fatty acid oxidation[4]. In a recent study, ETFB is ranked as one of the top proteins that have decreased protein level in AD human and 5xFAD mouse CSF and serum and human cortex samples[5]. In our transcriptome data, *ETFB*'s expression is lower in both AD and PSP in both TCx and CER. In summary, our qPCR data validated the RNAseq findings in our study.

IHC experiment of CXCR4 and ETFB

Immunohistochemical evaluation of CXCR4 immunostaining appeared glial in nature with morphologic evidence of microglial expression (Figure S3 A). CXCR4+ cells in an AD brain were found clustering within neuritic plaques (Figure S3 B), but not in the vicinity of basophilic structures consistent with neurofibrillary tangles (Figure S3 C). ETFB immunostaining was not robustly observed with diffuse staining of cortical neuropil. Granular staining was observed within the cytoplasm of some neurons in both AD and PSP cortex (Figure S4 aA,C). Evaluation of the white matter

revealed juxtanuclear cytoplasmic immunostaining within round nuclei morphologically consistent with oligodendrocytes (Figure S4 B,D).

Analysis of genes in Figure 3 using public epigenetics datasets

In Figure 3 of our manuscript, we depict the genes that are consistently up or down in both AD and PSP and that are enriched in the top GO processes. To determine whether these genes may be influenced by epigenetic mechanisms, we analyzed three available datasets (listed in Table S18) which comprise three types of epigenetic modifications, namely ATAC-seq, histone acetylation and methylation. The ATAC-seq dataset contains peaks (for neuron and glia cells separately) obtained from five individuals without history of psychiatric disorder[7]. ChIP-seq dataset contains differential H3K27ac, H3K9ac and H3K122ac peaks between AD and control subjects[6]. Methylation dataset contains Illumina BeadChip array data in AD and control subjects[8].

Our analyses of these three datasets provide evidence that the genes depicted in Figure 3 are likely subjective to epigenetic modification. Our main findings are as follows: Figure 3 genes, which have conserved expression changes in AD and PSP brains tend to (1) have broad promoter ATAC-seq peaks in glia cells. (2) gain H3K27ac and H3K9ac whereas lose H3K122ac in AD. (3) be hyper-methylated in AD samples compared to normal samples. We discuss these findings below.

Evidence of epigenetic modification for Figure 3 genes

We annotated genes of Figure 3 using the three public datasets and summarized in Table S19. Briefly, a gene gets a score=1 for histone modification (H3K9ac or H3K27ac or H3K122ac) if it is the nearest gene of a differential histone acetylation peak between AD and control samples. A gene gets score=1 for ATAC_glia (or ATAC_Neuron) if there is a glial (or neuronal) ATAC-seq peak in the promoter or 5'UTR region of this gene. A gene gets score=1 for methylation if there is a differentially methylated CpG probe between AD and control subjects within the gene body, or transcription start site (TSS) or untranslated region (UTR) of this gene. As Table S19 shows, out of 93 genes from Figure 3, which are in one or more of the categories shown, 84 or 90% genes has score 1 or more, 36 or 39% genes has score 3 or more. This demonstrates that most of these genes are under the influence of at least one type and >1/3 of them under the influence of >3 epigenetic modifications.

Evidence of glial broad promoter ATAC-seq peaks for Figure 3 genes

The ATAC-seq dataset in Table S18 contains open chromatin peaks obtained from ATAC-seq assay of 5 individuals for 14 brain regions and 2 cell types (neurons and non-neurons)[7]. We kept peaks that belong to the promoter or 5'UTR of all genes included in main text of this study[7]. We compared the peak size from Figure 3 genes in our study (GOI) vs. all other genes. As illustrated in supplementary Figure S5 and Table S20, the Wilcoxon rank sum one-sided test shows that in non-neuronal cells, i.e. mostly glial cells, peaks of genes of interest are significantly broader than those in all other genes (p-value = 4.0E-04). However, this difference is not observed in neurons (p-value=1.7E-01). Interestingly, among the genes associated with the broadest ATAC-seq peaks are those highlighted in Figure 3, such as methyl-binding protein (*MECP2*), methyltransferase (*KMT2D*), acetyltransferase (*KAT2B*), and helicase (*DHX9* and *WRN*). These findings demonstrate evidence of chromatin modification in glial cells for the genes shown in Figure 3.

Evidence of histone modifications for Figure 3 genes in AD

The ChIP-seq dataset (Table S18) contains peak comparison results of H3K27ac, H3K9ac and H3K122ac between AD patients (N=11) and old control (N=10) brain samples. We identified differential peaks ($p<0.05$) between AD and old control whose nearest gene is among Figure 3 genes; and checked how many are gain and how many are loss of signal (Table S21). We found that for H3K9ac and H3K29ac, the number of signal-gain peaks are ≥ twice the number of signal-loss peaks, whereas for H3K122ac the number of signal-loss peaks is twice the number of signal-gain peak. These findings demonstrate evidence of histone acetylation modifications in AD for the genes shown in Figure 3.

Evidence of methylation changes for Figure 3 genes in AD

The methylation BeadChip array dataset (Table S18) are from post-mortem DLPFC (dorsolateral prefrontal cortex)[8] of 315 AD and 221 control samples. Wilcoxon rank sum test was performed to compare AD and normal samples. Next, we extracted probes within or close to genes listed in Figure 3. Of the 1664 resulting probes, 30 had q values < 0.05. Interestingly, 29 out of these 30 probes (16 unique genes) have higher average methylation ratio in AD samples than that in normal samples (Table S22). These observations suggest that our genes in Figure 3 tend to be hyper-methylated in AD samples compared to normal samples in DLPFC.

Methods

Subjects and Samples

The study dataset has been made available to the research community and described in detail previously[9,10]. Briefly, AD, PSP and control subjects were diagnosed neuropathologically at autopsy. AD subjects are from the Mayo Clinic Brain Bank, had definite neuropathologic diagnosis according to the NINCDS-ADRDA criteria[11] and had Braak neurofibrillary tangle (NFT) stage of ≥4.0. All PSP subjects are from the Mayo Clinic Brain Bank and were diagnosed according to NINDS neuropathologic criteria[12]. Control subjects, either from Mayo Clinic Brain Bank or Banner Sun Health Institute, had Braak NFT stage of 3.0 or less, CERAD neuritic and cortical plaque densities of 0 (none) or 1 (sparse) and lacked various pathologic diagnoses. TCx and CER samples underwent RNA extractions via the Trizol/chloroform/ethanol method, followed by DNase and Cleanup of RNA using Qiagen RNeasy Mini Kit and Qiagen RNase -Free DNase Set. The quantity and quality of RNA samples were determined by the Agilent 2100 Bioanalyzer using the Agilent RNA 6000 Nano Chip. Samples included in this study all have RIN ≥5.0. Among final samples included in this study (231 TCx samples and 224 CER samples), 197 TCx and 197 CER samples were paired, i.e. from the same 197 subjects.

RNA sequencing

Library preparation and sequencing of the samples were conducted at the Mayo Clinic Genome Analysis Core using TruSeq RNA Sample Prep Kit (Illumina, San Diego, CA). The library was sequenced on Illumina HiSeq2000 instruments, generating 101 base-pair, paired-end raw reads. Raw reads were processed through MAPR-Seq pipeline[13] v1.0 which removed reads of low base-calling Phred scores, aligned remaining reads to human reference genome build GRCh37 using Tophat v2.0.12[14,15], counted reads in genes using Subread 1.4.4[16], and obtained QC measures from both pre-alignment reads and post-alignment reads using RSeQC toolkit[17,18] and fastQC (https://www.bioinformatics.babraham.ac.uk/projects/fastqc/). Samples that have high RNA degradation, or low reads mappability, or inconsistency between recorded sex and estimated sex using RNAseq chromosome Y expression were removed from downstream analysis. Raw reads of remaining samples were normalized using R cqn package[19] which took

into consideration library size, gene GC content and gene coding length, resulting in normalized expression in log2 scale. Additional information could be found in our previous publication[10,20].

Regression analysis

Multiple linear regression (MLR) were performed for each gene using normalized gene expression as dependent variable, diagnosis as primary independent variable, and RIN, age at death, sex, source of samples and flowcell as covariates (simple model), plus expression of five cell type markers (*ENO2* for neuron, *CD68* for microglia, *OLIG2* for oligodendrocyte, *GFAP* for astrocyte and *CD34* for endothelial cells) as covariates (comprehensive model), as previously published[10]. Diagnosis groups in these MLR were TCx ADvC, TCx PSPvC, CER ADvC and CER PSPvC.

Using β coefficients of DEGs of q-value<=1 (namely, <0.1, 0.05, 0.01) from the above MLR, simple linear regression was performed. Slopes and R^2 were obtained (Figure 1-2) from the following models: β.TCx.PSPvsCtrl ~ 1 + slope * β.TCx.ADvsCtrl, β.CER.PSPvsCtrl ~ 1 + slope * β.CER.ADvsCtrl, β.CER.ADvsCtrl ~ 1 + slope * β.TCx.ADvsCtrl, and β.CER.PSPvsCtrl ~ 1 + slope * β.TCx.PSPvsCtrl.

GO enrichment analysis

Differentially expressed genes were analyzed for GO enrichment using FUMA GWAS web server at https://fuma.ctglab.nl with MSigDB v7.0[21,22]. Background genes (N=14662) were the expressed coding genes in both TCx and CER cohorts, genes of interest were DEGs of q-value < 0.1 in both group comparisons and consistent in direction of expression change. Figures were made using R software environment.

Heatmaps and Volcano Plots

We scaled gene expression across samples (so that higher expressed genes have the same range of expression as lower expressed genes across samples), averaged the scaled expression per diagnosis group, and made heatmap plots. In addition to heatmaps, we made volcano plots for highlighted genes in main Figure 1 and 2. A typical volcano plot has -log10(q-value) (or transformed-q) on the y-axis. However, as we aligned data points from two comparisons, we standardized the transformed-q by computing (transformed-q - minimum(transformed-q))/maximum(transformed-q). Following this, we plotted β coefficient on the x-axis and the standardized transformed-q on y-axis.

Proteomics

To determine concordance of transcriptome changes with the proteome, we downloaded data from https://www.synapse.org/#!Synapse:syn18689335, which has results of differentially expressed protein/peptides from Liquid Chromatography Coupled to Tandem Mass Spectrometry (LC-MS/MS). The TCx protein results are from 84 AD and 31 controls, of which 80 AD and 25 controls are in this transcriptome study. The dataset contains about 5,000 proteins among which ~4000 corresponding genes are also in this transcriptome study. Proteome data for PSP on TCx (82 PSP vs 25 control) were downloaded from https://www.synapse.org/#!Synapse:syn9637748.

Validation of *CXCR4, SFRP2* and *ETFB* expression

To validate the expression levels of three genes selected from the RNAseq data based on their concordant expression changes in AD and PSP (*CXCR4*, *SFRP2* and *ETFB*), their mRNA were measured from TCx of autopsied patients with AD (n=10), PSP (n=10), and pathologically normal controls (n=10). These were a subset of the samples in our RNAseq dataset. Total RNA was extracted using TRIzol Reagent (Ambion Life Technology) followed by DNase cleanup step using RNeasy (Qiagen). The quality and quantity of these samples were determined by Agilent 2100 Bioanalyzer using an Agilent 6000 Nano Chip. Complementary DNA (cDNA) was synthesized from 400 ng of RNA, per sample, with Applied Biosystems High-Capacity cDNA Archive Kit was used as a template for relative quantitative PCR using ABI TaqMan chemistry (Applied Biosystems). Expression of these genes was quantified with TaqMan Probes Hs00607978_s1 (*CXCR4*), Hs00293258_m1 (*SFRP2*), and Hs01085511_m1 (*ETFB*) as well as Hs99999905_m1 (*GAPDH*) which was used as an endogenous control for global normalization. Each sample was run in 3 replicates on a QuantStudio 7 Real-Time PCR System.

IHC experiments of CXCR4 and ETFB

Immunohistochemistry (IHC) was performed to confirm and characterize the protein expression of two of the proteins with suitable antibodies, CXCR4 and ETFB. Sections from paraffin embedded tissue were cut at a thickness of 5 microns and allowed to dry overnight in a 60 degree oven. Following deparaffinization and rehydration, antigen retrieval was performed by steaming the sections for 30 minutes in either citrate buffer pH6 or Tris/EDTA buffer pH9. IHC staining was performed on the Thermo Scientific Autostainer 480S as follows: Endogenous peroxidase was blocked

for 5 minutes with 0.03% hydrogen peroxide. Sections were then treated for 20 minutes with 5% normal goat serum. Subsequently, sections were incubated for 45 minutes at room temperature in the following primary antibodies: Rabbit monoclonal anti-CXCR4, 1:500 (Abcam, [UMB2] ab124824), Rabbit polyclonal anti-ETFB, 1:250 (Invitrogen, PA5-106327). Following primary antibody incubation, the sections were incubated for 30 minutes at room temperature in Dako Envision+System- HRP Anti-Rabbit labelled Polymer, (Agilent Technologies, Inc., Santa Clara, CA, K4003). Peroxidase labeling was visualized with Dako Liquid DAB+ Substrate Chromogen System, (Agilent Technologies, Inc., Santa Clara, CA, K3468). The sections were then counterstained with Gill 1 Hematoxylin (Thermo Scientific, 6765006), and coverslipped with Cytoseal™ XYL mounting medium, (Thermo Scientific, 8312-4).

ATAC-seq peak analysis

ATAC-seq dataset was downloaded from bendlj01.u.hpc.mssm.edu/multireg/. This dataset contains open chromatin regions obtained from ATAC-seq assay from 5 individuals of 14 brain regions and 2 cell types (neurons and non-neurons)[7]. Among the 14 brain regions, STC (superior temporal cortex) is the same region as TCx region in our study. Therefore, we investigated the open chromatin peaks called in STC, for neurons and non-neurons separately. The peak calling approach could be found in original publication[7]. We kept peaks that belong to the promoter or 5'UTR of all genes included in main text (i.e., the 14662 in Table1 of main text of the ATAC-seq study[7]). We sought to determine whether the genes in Figure 3 of the main text had evidence of chromatin modifications. Figure 3 genes are those that are most frequently involved in top enriched GO terms in our study. These GO terms were obtained from genes that are consistently up or down between AD vs. control and PSP vs control in TCx or in CER. We compared the ATAC-seq peak size from Figure 3 genes to those from other genes using Wilcoxon rank sum one-sided test to determine whether the genes with conserved brain transcriptome changes in AD and PSP were under epigenetic control based on ATAC-seq data.

H3K27ac, H3K9ac and H3K122ac analysis

We also determined evidence of histone modification for the genes depicted in Figure 3. We downloaded a public ChIP-seq (chromatin immunoprecipitation sequencing) dataset of post-mortem lateral temporal lobe at GSE130746[6]. This dataset contains peak comparison results of H3K27ac, H3K9ac and H3K122ac between AD patients (N=11) and old control (N=10). The description of demographics, sample preparation, peak calling and peak comparison

between groups can be found in the original publication[6]. Briefly, peaks were called with MACS2 for each group and consensus peaks were obtained using MTL method. The ChIP-seq signal in log2 RPKM (per kilobase of transcript, per million mapped reads) in consensus peaks were obtained for each individual and were compared between AD and old control groups using two-sided Wilcoxon rank-sum test.

Methylation analysis

To identify evidence of differential methylation for the genes shown in Figure 3, we downloaded a methylation data set at Sage Synapse portal at www.synapse.org on May 25, 2021. This dataset contains Illumina HumanMethylation450 BeadChip array of post-mortem DLPFC (dorsolateral prefrontal cortex)[8]. A description of data generation and data QC is provided at www.synapse.org/#!Synapse:syn3157275. In this dataset, we identified 315 AD samples of Braak score>=4 and CERAD score=1 or 2, and 221 normal samples of Braak score <=3 and CERAD score=3 or 4. The explanation of these variables and recommendation of usage may be found at clinical code book in Table S1 of the original study[8]. The above definition of AD and normal samples based on pathology scores is consistent with our definition of AD and control samples in our main text analyses. Wilcoxon rank sum test was performed for probes within or close to genes included in main text in Table1 of the manuscript for this methylation data[8]. AD samples were compared to normal samples, and p-values and q-values were obtained for differential methylation.

Study approval

This study was approved by the Mayo Clinic institutional review board. Informed consent was obtained from the participants or their next-of-kin as applicable.

Data access

The access information of transcriptomic data of TCx and CER and proteomics data is listed in supplementary table S1. The access information of epigenomic datasets is listed in supplementary table S18.

Acknowledgements

The transcriptomic results published here are in whole or in part based on data obtained from the AD Knowledge Portal (https://adknowledgeportal.org). The Mayo RNAseq study data was led by Dr. Nilüfer Ertekin-Taner, Mayo Clinic, Jacksonville, FL as part of the multi-PI U01 AG046139 (MPIs Golde, Ertekin-Taner, Younkin, Price). Samples were provided from the following sources: The Mayo Clinic Brain Bank. Data collection was supported through funding by NIA grants P50 AG016574, R01 AG032990, U01 AG046139, R01 AG018023, U01 AG006576, U01 AG006786, R01 AG025711, R01 AG017216, R01 AG003949, NINDS grant R01 NS080820, CurePSP Foundation, and support from Mayo Foundation. Study data includes samples collected through the Sun Health Research Institute Brain and Body Donation Program of Sun City, Arizona. The Brain and Body Donation Program is supported by the National Institute of Neurological Disorders and Stroke (U24 NS072026 National Brain and Tissue Resource for Parkinsons Disease and Related Disorders), the National Institute on Aging (P30 AG19610 Arizona Alzheimers Disease Core Center), the Arizona Department of Health Services (contract 211002, Arizona Alzheimers Research Center), the Arizona Biomedical Research Commission (contracts 4001, 0011, 05-901 and 1001 to the Arizona Parkinson's Disease Consortium) and the Michael J. Fox Foundation for Parkinsons Research.

The proteomics results published here are in whole or in part based on data obtained from the AD Knowledge Portal (https://adknowledgeportal.org). These data were provided by Dr. Levey from Emory University based on postmortem brain tissue provided by The Mayo Clinic Brain Bank. Data collection was supported through funding by NIA grants P50 AG016574, R01 AG032990, U01 AG046139, R01 AG018023, U01 AG006576, U01 AG006786, R01 AG025711, R01 AG017216, R01 AG003949, NINDS grant R01 NS080820, CurePSP Foundation, and support from Mayo Foundation.

The methylation results published here are in whole or in part based on data obtained from the AD Knowledge Portal (https://adknowledgeportal.org). Study data were provided by the Rush Alzheimer's Disease Center, Rush University Medical Center, Chicago. Data collection was supported through funding by NIA grants P30AG10161 (ROS), R01AG15819 (ROSMAP; genomics and RNAseq), R01AG17917 (MAP), R01AG30146, R01AG36042 (5hC methylation, ATACseq), RC2AG036547 (H3K9Ac), R01AG36836 (RNAseq), R01AG48015 (monocyte RNAseq) RF1AG57473 (single nucleus RNAseq), U01AG32984 (genomic and whole exome sequencing), U01AG46152 (ROSMAP AMP-AD, targeted

proteomics), U01AG46161(TMT proteomics), U01AG61356 (whole genome sequencing, targeted proteomics, ROSMAP AMP-AD), the Illinois Department of Public Health (ROSMAP), and the Translational Genomics Research Institute (genomic). Additional phenotypic data can be requested at www.radc.rush.edu.

References

1. Bonham, L.W., *et al.* CXCR4 involvement in neurodegenerative diseases. *Translational Psychiatry* **8**, 73 (2018).
2. Lin, H., *et al.* sFRP2 activates Wnt/β-catenin signaling in cardiac fibroblasts: differential roles in cell growth, energy metabolism, and extracellular matrix remodeling. *American Journal of Physiology-Cell Physiology* **311**, C710-C719 (2016).
3. Esteve, P., *et al.* SFRPs act as negative modulators of ADAM10 to regulate retinal neurogenesis. *Nature Neuroscience* **14**, 562-569 (2011).
4. Li, Y., *et al.* Functional characterization of electron-transferring flavoprotein and its dehydrogenase required for fungal development and plant infection by the rice blast fungus. *Scientific Reports* **6**, 24911 (2016).
5. Wang, H., *et al.* Integrated analysis of ultra-deep proteomes in cortex, cerebrospinal fluid and serum reveals a mitochondrial signature in Alzheimer's disease. *Molecular Neurodegeneration* **15**, 43 (2020).
6. Nativio, R., *et al.* An integrated multi-omics approach identifies epigenetic alterations associated with Alzheimer's disease. *Nature Genetics* **52**, 1024-1035 (2020).
7. Fullard, J.F., *et al.* An atlas of chromatin accessibility in the adult human brain. *Genome Res* **28**, 1243-1252 (2018).
8. De Jager, P.L., *et al.* Alzheimer's disease: early alterations in brain DNA methylation at ANK1, BIN1, RHBDF2 and other loci. *Nat Neurosci* **17**, 1156-1163 (2014).
9. Allen, M., *et al.* Human whole genome genotype and transcriptome data for Alzheimer's and other neurodegenerative diseases. *Scientific Data* **3**, 160089 (2016).
10. Allen, M., *et al.* Conserved brain myelination networks are altered in Alzheimer's and other neurodegenerative diseases. *Alzheimer's & Dementia* (2017).
11. McKhann, G., *et al.* Clinical diagnosis of Alzheimer's disease: report of the NINCDS-ADRDA Work Group under the auspices of Department of Health and Human Services Task Force on Alzheimer's Disease. *Neurology* **34**, 939-944 (1984).
12. Hauw, J.J., *et al.* Preliminary NINDS neuropathologic criteria for Steele-Richardson-Olszewski syndrome (progressive supranuclear palsy). *Neurology* **44**, 2015-2019 (1994).
13. Kalari, K.R., *et al.* MAP-RSeq: Mayo Analysis Pipeline for RNA sequencing. *BMC Bioinformatics* **15**, 224 (2014).
14. Trapnell, C., Pachter, L. & Salzberg, S.L. TopHat: discovering splice junctions with RNA-Seq. *Bioinformatics* **25**, 1105-1111 (2009).
15. Trapnell, C., *et al.* Differential gene and transcript expression analysis of RNA-seq experiments with TopHat and Cufflinks. *Nature Protocols* **7**, 562 (2012).
16. Liao, Y., Smyth, G.K. & Shi, W. The Subread aligner: fast, accurate and scalable read mapping by seed-and-vote. *Nucleic Acids Research* **41**, e108-e108 (2013).
17. Ashton, R.S., *et al.* Astrocytes regulate adult hippocampal neurogenesis through ephrin-B signaling. *Nature Neuroscience* **15**, 1399 (2012).
18. Wang, L., Wang, S. & Li, W. RSeQC: quality control of RNA-seq experiments. *Bioinformatics* **28**, 2184-2185 (2012).
19. Hansen, K.D., Irizarry, R.A. & Wu, Z. Removing technical variability in RNA-seq data using conditional quantile normalization. *Biostatistics* **13**, 204-216 (2012).
20. Allen, M., *et al.* Divergent brain gene expression patterns associate with distinct cell-specific tau neuropathology traits in progressive supranuclear palsy. *Acta Neuropathol* **136**, 709-727 (2018).
21. Watanabe, K., Taskesen, E., van Bochoven, A. & Posthuma, D. Functional mapping and annotation of genetic associations with FUMA. *Nature Communications* **8**, 1826 (2017).
22. Liberzon, A., *et al.* Molecular signatures database (MSigDB) 3.0. *Bioinformatics (Oxford, England)* **27**, 1739-1740 (2011).

Supplementary Figures

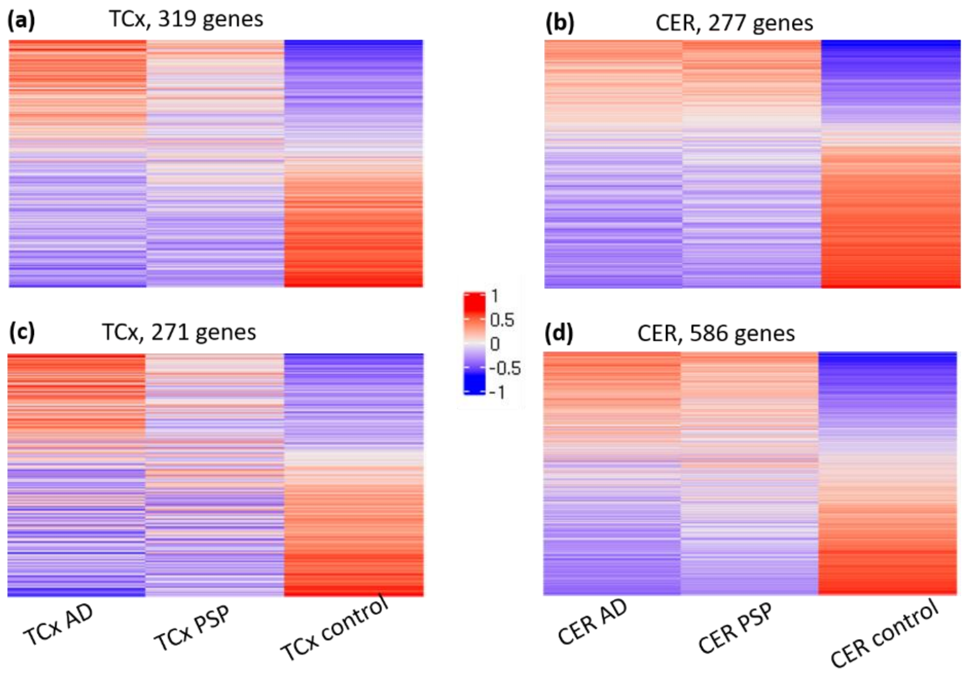

Figure S1. Heatmaps of averaged gene expression of genes from (a) Figure 1A. (b) Figure 1B. (c) Figure 1C. (d) Figure 1D. The expression of these genes are significantly different both between AD and control, and between PSP and control in TCx or CER based on linear regression analysis with simple model (a and b) or comprehensive model (c and d).

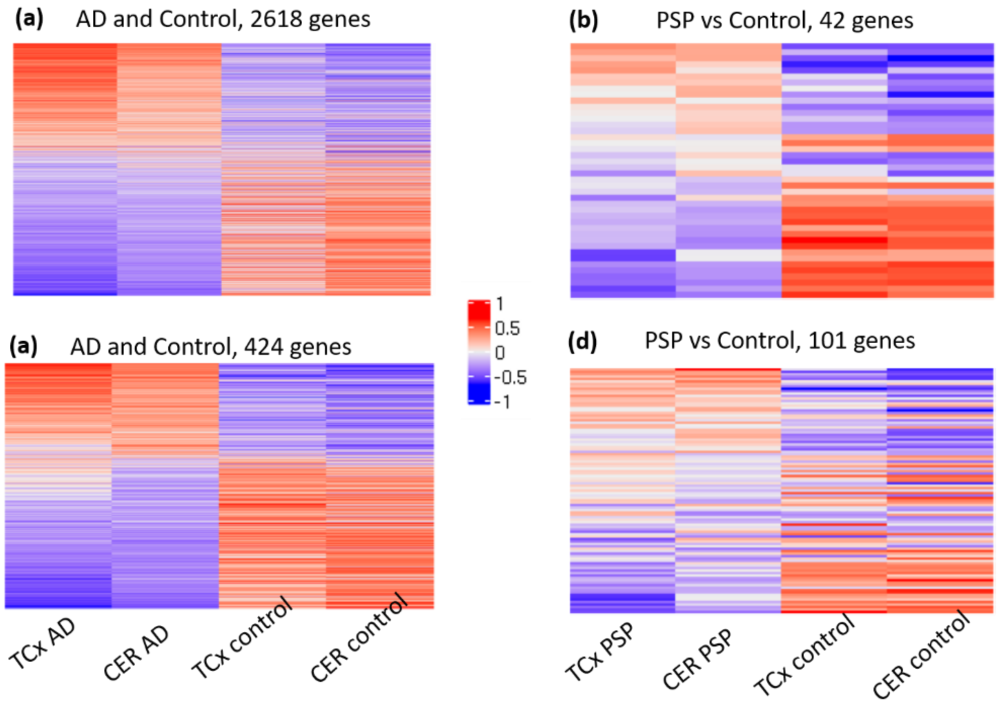

Figure S2. Heatmaps of averaged gene expression of genes from (a) Figure 2A. (b) Figure 2B. (c) Figure 2C. (d) Figure 2D. These expression of these genes are significantly different between AD (or PSP) and control in both TCx and CER based on linear regression analysis with simple model (a and b) or comprehensive model (c and d).

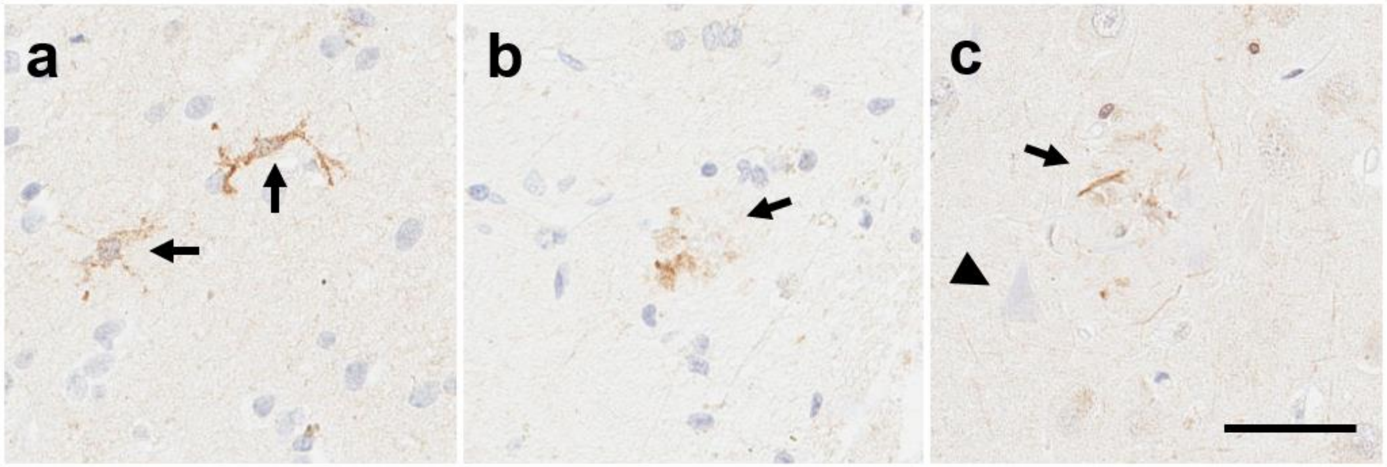

Figure S3. CXCR4 immunostaining. (a) CXCR4 immunostaining was found in a subset of glial cells with the morphologic appearance of microglia (arrows) in an AD parahippocampal cortex. In an AD temporal cortex (b) and CA1 of hippocampus (c), clustering of CXCR4+ cells and fibers were observed in structures consistent with neuritic plaques (arrow), but did not appear in the vicinity of basophilic neurofibrillary tangles (arrowhead). Scale bar 50 µm. (AD=Alzheimer’s disease, 74 year old female).

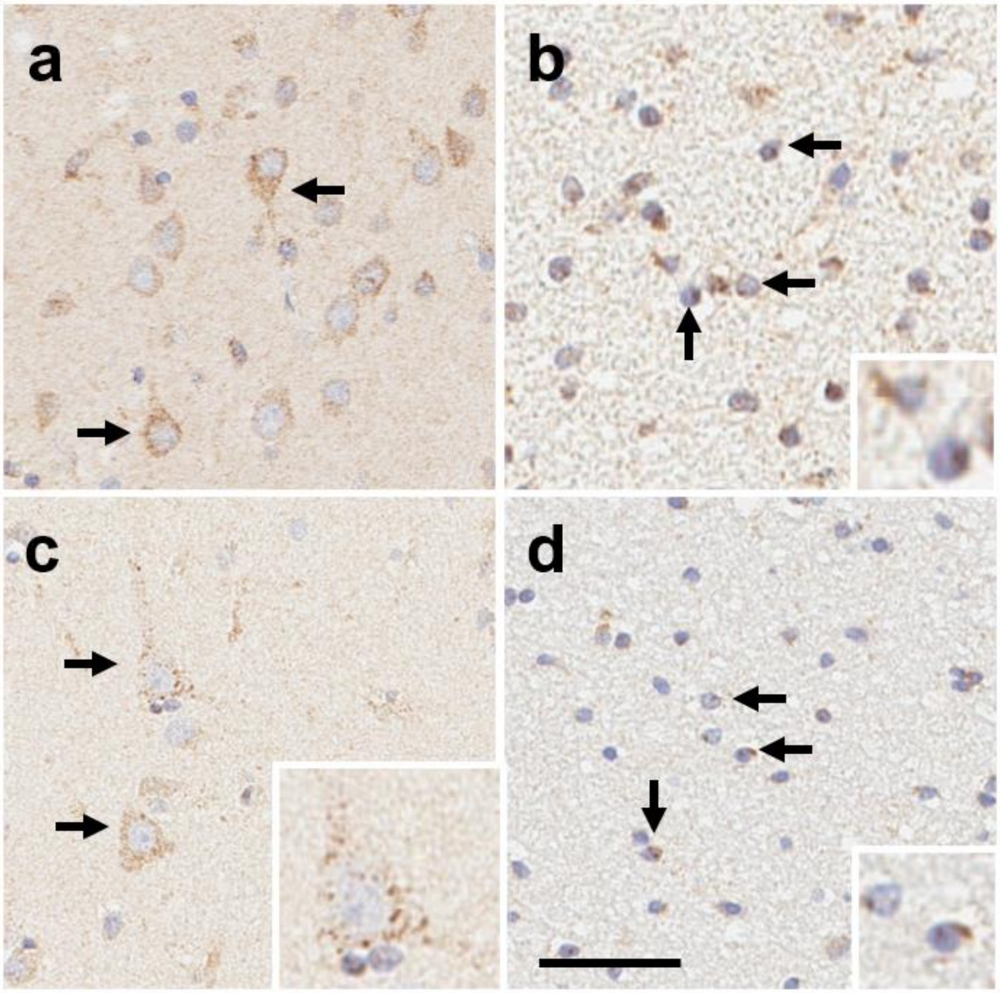

Figure S4. ETFB immunostaining. ETFB was weakly detected in both (a,c) neurons in temporal cortex and oligodendrocytes (b,d) in temporal lobe. A subset of cortical neurons in (a) AD and (c) PSP were observed to have ETFB immunoreactivity in the cytoplasm that was granular in appearance (inset). The white matter in (b) AD and (d) PSP was identified to have granular ETFB immunoreactivity in juxtanuclear cytoplasm of cells with morphologic appearance of oligodendrocytes. Scale bar 50 um (AD=Alzheimer's disease, 85 year old female; PSP=Progressive supranuclear palsy, 65 year old female).

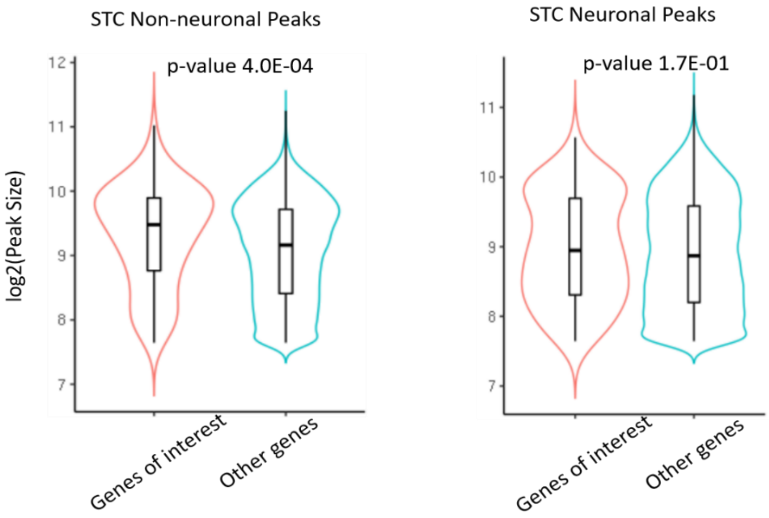

Figure S5. Distribution of peak sizes. (left): peaks called from glia (non-neuron) cells. (right): peaks called from neurons. P values are from one-sided Wilcoxon rank sum test.
